# Oriented Electric Fields—Universal Catalysts

**DOI:** 10.1021/acs.accounts.5c00508

**Published:** 2025-09-25

**Authors:** Sason Shaik, David Danovich, Surajit Kalita, Kshatresh D. Dubey

**Affiliations:** † Institute of Chemistry, 26742 The Hebrew University of Jerusalem, Jerusalem 9190401, Israel; ‡ Department of Chemistry, School of Natural Sciences, 310513 Shiv Nadar Institution of Eminence, 201314 Delhi NCR, India

## Abstract

This Account outlines principles of electric-field-mediated chemistry, whereby oriented-external electric fields (OEEFs) function as universal “reagents” that control reactivity/selectivity and structures of molecules/clusters. The TOC graphics illustrate the rate-enhancing OEEFs for two different reactions. For the Diels–Alder reaction, we also mark the corresponding reaction-axis (**RA**). The **RA** arrow specifies the **directional-flow, of the electron density and bond-coupling**, from reactants to products (**RC**→**PC**). Determining the **RA** direction, for a given process, involves curly arrow-pushing in the charge-transfer direction. By convention, the arrowhead of the **RA signifies the direction of the negative charge flow** (Scheme 2). The arrowhead of the ***F***_**Z**_ (OEEF) vector is marked as positive, hence corroborating the direction of negative charge flow, which will be induced by ***F***_**Z**_.

Thus, as the Account demonstrates, *the impact of OEEFs on reactions and structural transformations is unique*. **Energy-barriers-lowering generally occurs along a single direction in space**, specified by the **RA**. Furthermore, **the OEEF also catalyzes reactions in the presence of solvents**! For example, the computed OEEF lowers the barrier of the Menshutkin reaction (pyridine/CH_3_–I) by 10.6–12.6 kcal/mol in the three polar solvents. Thus, **solvent screening of the OEEF is imperfect** *(see F*_*solvent‑induced*_ *in the conspectus art)*, and hence, chemical reactions are not limited to gas- or solid-phases. As the main text elaborates, **this imperfect screening-effect in solution is fundamental**, and applicable to reactions and to OEEF-induced structural changes. **As such, the OEEF is a universal enhancer of chemical change**.

The Account starts with conceptual principles for understanding and predicting the theoretically computed and/or experimentally observed OEEF effects on chemical reactions as well as structural transformations. These principles highlight the role of OEEFs as **tweezers** ***that orient molecular species*** **along the respective RAs**, and accelerate their transformation to products.

Subsequently, the paper describes experimental support of the theoretical results and guidelines. **Some of the applications also use continuous-flow setups, which** ***will eventually scale-up product yields to Molar concentrations***, and render *OEEFs as practical tools in chemistry*. Evidence is presented for the potential existence of **OEEF/thermal dichotomy**, wherein **the OEEF-induced products differ from those which are produced corresponding thermal-only reactions** (see later work by Matile et al.).

The paper addresses also an important structural process; on the type of EEF (oscillating vs static), which carries out most effectively the decomposition of peptide-plaques (e.g., as those which are found in brains during Alzheimer’s disease). We show that in accord with experimental results, the most efficient decomposition is incurred with oscillating EEFs in the frequency range that is smaller than or equal to 1 GHz.

The article concludes with a vision that in the near future, ***OEEF usage will change chemical education, chemical practice, and the art of making molecules***.

## Graphical abstract

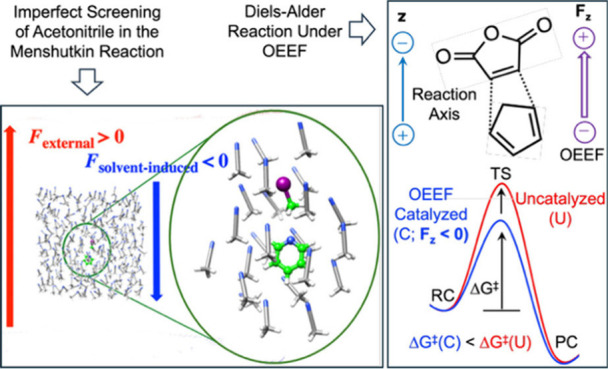

## Key References

-

Shaik, S. My Vision of the Future of Electric-Field-Aided Chemistry in 2050. ACS Phys. Chem. Au 2024, 4 (3), 191–201 38800723 10.1021/acsphyschemau.3c00064PMC11117677.^1^ *This publication highlights the vision of oriented-external-electric-Fields (OEEFs) as* **universal catalysts** *. OEEFs along the reaction* *axis* (**RA**) *orient the reactants, increase transition states’ (TSs) dipole-moments, and lower barriers, proportionally to the product OEEF*x*TS dipole moment*.
-

Wang, Z. ; Danovich, D. ; Ramanan, R. ; Shaik, S. Oriented-External Electric Fields Create Absolute Enantioselectivity in Diels–Alder Reactions: The Importance of the Molecular Dipole Moment. J. Am. Chem. Soc. 2018, 140, 13350–13359.30232877 10.1021/jacs.8b08233 ^2^ *The study demonstrates that OEEFs along the C=C bond of dienophiles, which react with a given diene,* **lead to enantioselective Diels–Alder products**. *The enantioselectivity range (3–30 kcal/mol) increases in proportion to the dipole moment of the dienophile.*
-

Dubey, K. D. ; Stuyver, T. ; Kalita, S. ; Shaik, S. Solvent-Reorganization and Rate-Regulation of a Menshutkin Reaction by Oriented-External Electric Fields Are Revealed by Combination of MD and QM/MM Calculations. J. Am. Chem. Soc. 2020, 142, 9955–9965.32369357 10.1021/jacs.9b13029PMC7304904 ^3^ *This work demonstrates that solvent screening* **does not impair the EEF-induced rate-enhancement** *of Menshutkin reactions. In fact, the predicted energy-barrier lowering, in three polar solvents, amounts to 10.6–12.6 kcal/mol*.
-

Meir, R. ; Chen, H. ; Lai, W. ; Shaik, S. Oriented Electric Fields Accelerate Diels–Alder Reactions and Control the *endo*/*exo* Selectivity. ChemPhysChem 2010, 11, 301–310 19998402 10.1002/cphc.200900848.^4^ *The first study that demonstrated the OEEF-control of energy barrier-lowering and exo/endo products-ratios in the Diels–Alder reaction of maleic anhydride and cyclopentadiene. The impact increases proportionally to the product of the OEEF by the TS dipole-moment.*

## 1 Introduction

Electrostatic effects^5^ due to ions and dipoles, which exist in solvents,^6^ ionic liquids,^7^ and proteins (enzymes)^8–14^ and on surfaces, impact chemical-reactivity and molecular-structure-changes.^15^ The present ACR-article focuses on external electric fields (EEFs),^16–21^ and on designed EFs in a (short-circuited) electrochemical device,^22^ which are all applied onto molecules and affect thereby their molecular structures and reactivity/selectivity patterns.

As shown in Scheme 1, these EF sources can be generated by applying/creating voltage differences in devices, which are devoid of electric currents.^1–23^ Scheme 1a is a single-molecular device that performs S_N_2 reactions, while Schemes 1b and 1c can, in principle, contain macroscopic amounts of molecules which react and produce products.^21–24^ As such, these devices and similar ones function as **“EF reagents”** that alter molecular structures (e.g., azoarene isomerization^25^), reaction rates and selectivity patterns.^1–25^ Similarly, the recently designed **Oriented-EF (OEF) in a supramolecular cage**,^24^ was demonstrated to perform oxidations of molecules in a manner that resembles P450 enzymes.

**Scheme 1:**
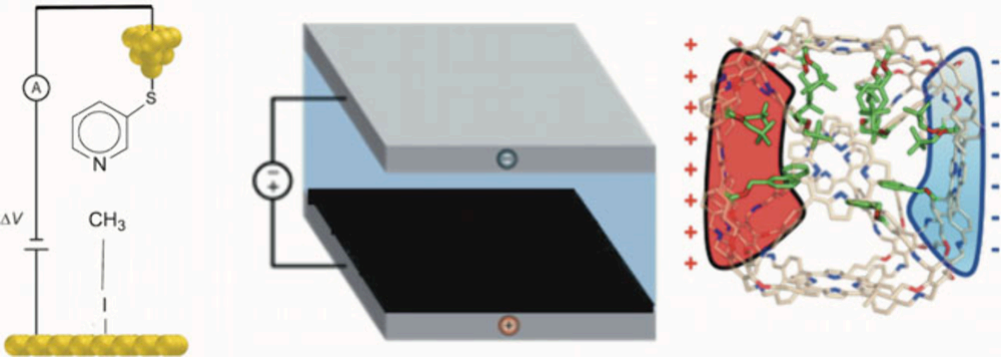
Devices for Electric-Field Applications: (a) a Bimolecular Cell That Uses Voltage Differences to Control an S_N_2 Reaction; (b) an Electrochemical Cell Wherein the Black-Painted Electrode Is Coated with an Insulator, and Hence Does Not Transmit Current, while Generating an OEEF Due to the Spatial Separation of the Opposite Ions of the Electrolyte in the Cell;^a^ (c) a Supramolecular Device Which Possesses Oppositely Charged Walls That Create an OEF, and a Built-in Iron-Porphyrin as a Reagent^24^

a Reproduced with permission from ref 22. Copyright 2013 American Chemical Society.

As shown later, when these electric fields are oriented (OEF, OEEF), they will initially cause polar molecules to get oriented in space,^24,26–28^ along the electric-field vector. The latter vector defines also the direction of the reaction axis/path,^1–4,28–37^ along which *the electron density and bond pairing change from reactant-like (* **R** *) to product-like (* **P** *) patterns,* and thereby catalyzes the reaction.

Note that an OEEF is an antisymmetric vector with respect to a mirror element of symmetry passing through the center of the OEEF arrow. Hence, the OEEF may, in principle, stimulate reactivity patterns that differ from those obtained under the common thermal conditions. In addition to reactivity and structure, the ACR article discusses oscillating external electric fields (Os-EEF), as restructuring tools that decompose peptide plaques.^38,39^

Let us first outline key patterns of electric fields’ effects,^1–4^ and then discuss some applications, and follow with experimental verifications by chemists who tested the electric field impact on structure and reactivity, in response to computational studies initiated by us and other groups.

## 2 Principles of Structure and Reactivity Patterns under OEEFs

### 2.1 The **RA** Rule: Its Significance and Applications

Having computed a variety of reactions,^1,3,4,32^ we demonstrated that the **OEEF affects energy barriers, primarily along a single direction**. This direction is the “reaction axis” (**RA**),^1,4,29–35^ which accounts for the transformation of the electron density and bond-pairing, from reactant- to product-like. As we show in Scheme 2 for a generic S_N_2 reaction, the **RA** can be traced by *applying* **curly arrow-pushing**. This simple mnemonic turns out to be a useful descriptor of the **R** → **P** transformation. Thus, as shown in Scheme 2, the **RA** is the vector that stretches, along axis Z, from N to R-X, and reveals that the reaction will be catalyzed by an OEEF along this axis (by F_Z_, Scheme 2). Let us follow with demonstrations that the computed/observed OEEF-effect is indeed primarily **unidirectional**.

**Scheme 2:**
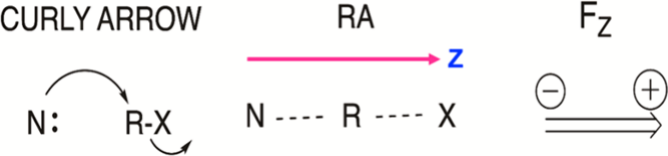
Reaction Axis (**RA**) and the Oriented-Electric Field Vector (*F*_Z_) in an S_N_2 Reaction

To avoid multilabels, the **RA** directions will uniformly be labeled as the **Z** axis, as in Scheme 2. The corresponding *F*_Z_ vector is drawn here with a particular convention, which is illustrated in Scheme 3.

**Scheme 3:**
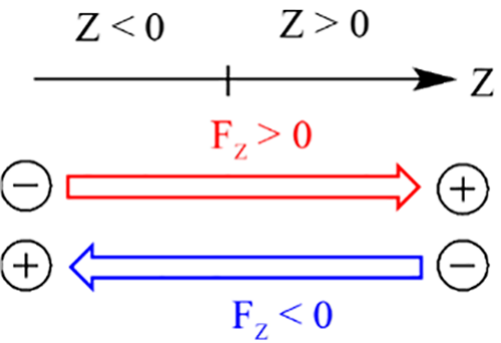
Convention used for positively and negatively oriented OEEFs along the *Z*-axis

As noted above, by this convention, the positive pole of the vector *F*_*Z*_ > 0 is the arrowhead in Scheme 3, whereas the arrow tail is the negative pole. The vector *F*_*Z*_ < 0 is oppositely oriented. This convention is adopted throughout this article.

Figure 1 exemplifies the **RA** rule by using a computed gas-phase Menshutkin S_N_2 reaction (of pyridine + CH_3_–I) under OEEF.^17,33^ **It is apparent that significant changes in reaction barriers occur along the** *Z* **-axis**, which is the corresponding **RA** of the reaction. The X and Y directions have little impact on the barriers.

**Figure 1:**
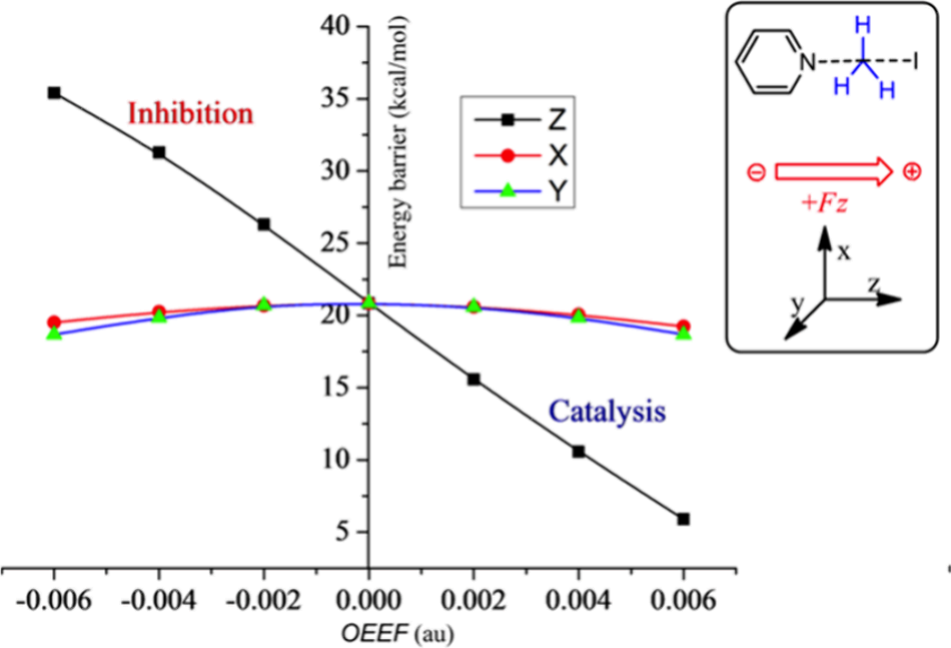
Variations of computed energy barriers (kcal/mol) for the Menshutkin reaction as a function of the OEEF (1 au = 51.4 V/Å) along the X, Y, and Z directions. The convention for *F*_Z_ > 0 is drawn in the inset to the right of the plot. Reproduced from Figure 1b in ref 33. Copyright 2018 American Chemical Society.

Thus, the application of *F*_Z_ > 0 lowers the barriers by ca. ∼ 20 kcal/mol, while flipping the field to *F*_Z_ < 0 raises the barriers by a similar amount. Hence, the OEEF along the direction N- - -C- - -I will have two regions; *F*_Z_ > 0 will cause barrier lowering, whereas *F*_Z_ < 0 will raise the barrier, and thereby inhibit the reaction.

As such, the **RA** rule is predictive, and this has been proven in many examples that we tested computationally. Indeed, recently Tang et al.^35^ tested this theoretical prediction and showed that the Menshutkin reaction (of substituted pyridine and alkyl iodide) is massively catalyzed by 39,000-fold in a single-molecule device, which fixes the oriented reactants under a voltage difference, and records the tunneling-current response^21,35^ during the reaction.

Note that the inhibitory section (*F*_Z_ < 0 in Figure 1) is not observable unless the reaction complex can **be forced into the N- - -C- - -I arrangement**, while simultaneously reversing the voltage direction in the reaction cell (i.e., single-molecular cells^17,21,35^ ). We display the computed repulsive-portion of the reactivity plot in Figure 1, along the Z axis, because this underscores the unidirectionality of the barrier lowering along the **RA**(Z). In this sense, Figure 1 is an archetype of reactivity under the OEEF.

### 2.2 Potential Effects of Symmetry

Being a vector, *F*_Z_ is antisymmetric with respect to a mirror image in the X,Y-plane. As such, it may affect reactivity by virtue of symmetry control. Let us then consider the frontside Menshutkin reaction, which is depicted in Scheme 4.^33^

**Scheme 4:**
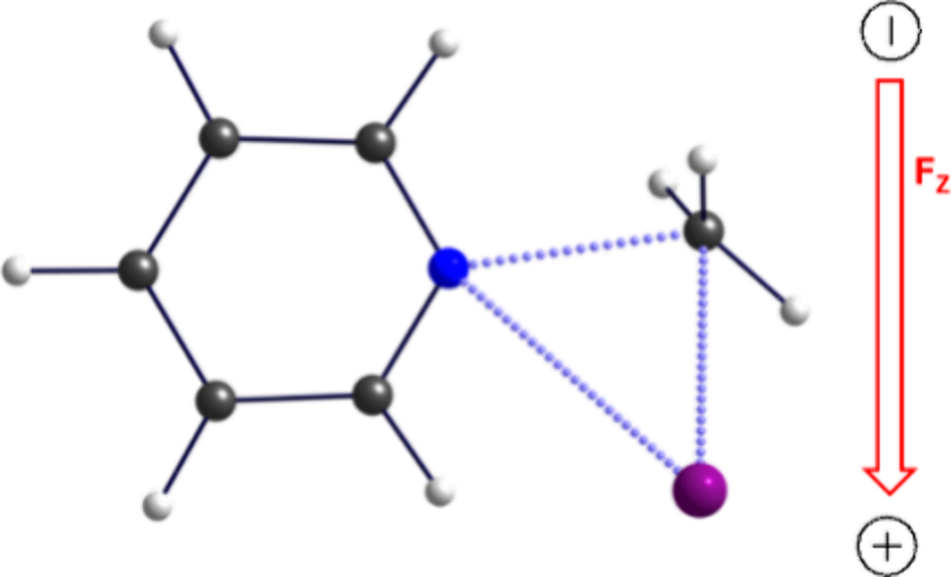
A Menshutkin Reaction in Which Pyridine (the N Atom Is Drawn in Blue) Attacks CH_3_I via the Frontside of the C–I Bond in the Presence of an OEEF^a^

a The OEEF vector is aligned along the C–I bond, such that the positive pole faces iodine, which will depart as an iodide anion.

Thus, since the σ* orbital of the C–I bond has a node in the *X*–*Y* plane that bisects the C–I bond, σ* is antisymmetric. Similarly, an OEEF vector along the C–I bond is antisymmetric with respect to the same mirror plane. As such, the combined symmetry of the σ*­(C–I) orbital and of the applied *F*_Z_, along the C–I bond, is symmetric. Similarly, the lone-pair of the attacking nitrogen (of pyridine) is symmetric with respect to the *X*–*Y* plane in Scheme 4. Therefore, in principle, applying the OEEF along the C–I bond while the pyridine attack is oriented toward the mid C–I bond will enable the **“allowed” front-side S**_**N**_ **2 displacement** (albeit with a high barrier, due to weaker TS bonds, compared with the backside path). An experimental test of this prediction can be achieved using a chiral alkyl iodide while linking the pyridine to a surface (so it cannot perform a backside attack on the alkyl iodide).

## 3 OEEF Tweezes Reactants in Space

As has been demonstrated by Friedrich,^26–28^ an OEEF is capable of orienting polar molecular-species along the OEEF axis and the respective molecular dipole moment. Hence, the OEEF creates the driving force for keeping two reactants along the **RA**. Figure 2 shows^36^ the halogen bond of NH_3_ and Cl_2_ in the presence of an OEEF. It is seen that the OEEF stabilizes the halogen bond by 25.3 kcal/mol against a 90° rotation (Figures 2b vs [Fig fig2]a). With such a significant interaction energy, it is clear that the halogen-bond complex will be steadfastly aligned along the OEEF-axis.

**Figure 2:**
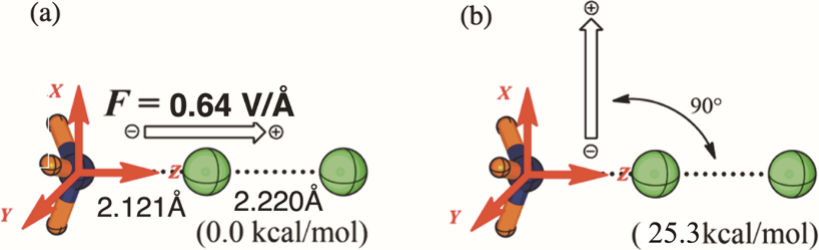
Computed**-**tweezing of H_3_N···Cl_2_ by an OEEF (*F*_Z_ = 0.64 V/Å). Cl_2_ is represented by the green spheres. (a) The interaction-energy of H_3_N···Cl_2_ with the aligned OEEF vector is 25.3 kcal/mol. (b) Thus, rotating the halogen bond complex by 90° must overcome a barrier of 25.3 kcal/mol. Reproduced from ref 36. Copyright 2019 American Chemical Society.

Since the halogen-bond complex is restricted by the OEEF, the field will also activate the halogen bond and cleave the Cl–Cl bond shown in Figure 3. Note that the reaction barrier is lower than the rotational barrier in Figure 2 (as found, a linear arrangement of the halogen-bond complex is maintained along the reaction coordinate).^36^

**Figure 3:**
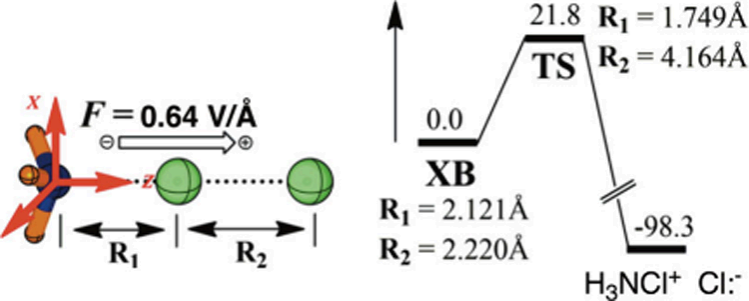
Computed gas-phase reaction barrier for the halogen bond (**XB**) cleavage to H_3_NCl^+^ and Cl^–^. Reproduced from ref 36. Copyright 2019 American Chemical Society.

Such a catalyzed halogen-bond cleavage was recently confirmed by experiment^37^ using the intrinsic water-droplet’s EF, which accelerates the rate of cleavage of halogen-bonds. Thus, water droplets are considered to exert EFs^40,41^ perpendicular to the surface of the droplet, and these EFs appear to catalyze a variety of reactions, including formation of H_2_O_2_ in air, etc.^41^ Generally speaking, hydrogen-bonded water molecules have higher positive charges on the inner H atom, that may serve as a local EF,^42^ that enhances reactions, as demonstrated in the study of ammonium hydroxide formation in water.^43^

## 4 Estimating Energetic Effects of OEEFs

How does one assess the magnitudes of electric fields? Given a charge distribution of molecular entities, the respective EF vector can be computed using the software packages TITAN^15^ and TUPA.^44^ Once the EF is so-determined, usage of the dipole moment (μ) of the molecular entity enables one to determine the EF-dipole interaction energy, using the equation (eq 1) derived by Lockhart/Fried and Boxer.^45–47^

Thus, in their work on Ketosteroid isomerase (KSI) enzymes, Fried and Boxer^46,47^ demonstrated how to quantify the local electric field in enzymes by use of vibrational-Stark-effect spectroscopy. They reported that the native KSI enzyme applies a substantial EF (*F*) of 1.44 V/Å on the carbonyl group of steroids, which enhances the KSI reactivity. They further formulated the direct-product expression, in eq 1, for the interaction between the two vectors: the electric field (*F* in V/Å units) of the enzyme, and the dipole moment (μ in Debye) of the reacting molecule:

1 $$\Delta E\;\left(\mathrm{kcal}/\mathrm{mol}\right)=4.8\overset{⃗}{F\cdot }\mathrm{\mu ⃗}$$

When the two vectors are aligned along the same axis, the respective interaction energy, Δ*E*, is a simple product of the vectors. These interaction energies can be quite large; for example, an OEEF of 0.64 V/Å (Figure 2) restricts the halogen bond along the OEEF axis, with a rotational-barrier of 25.3 kcal/mol (Figure 2).^36^

Another example is the impact of OEEF on the regioselectivity of propene oxidation by a model of the oxo-iron active species of cytochrome P450. The oxidation can take place either on the allylic C–H or on the C=C moieties of propene,^29^ and with the directional field-preferences indicated in Figure 4.

**Figure 4:**
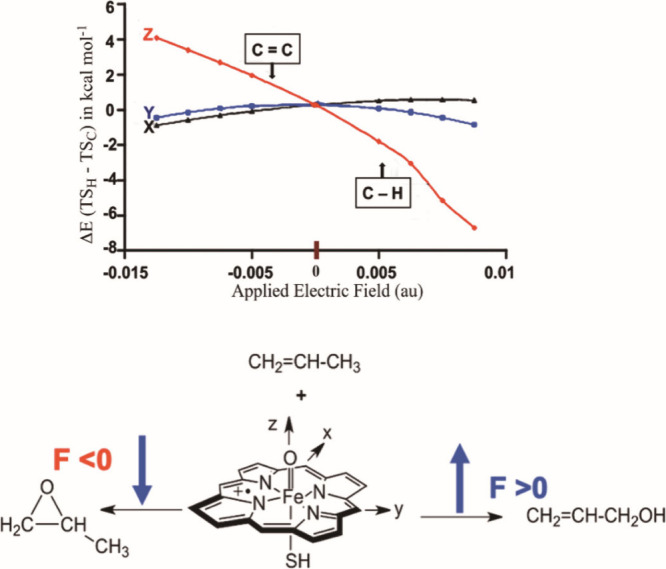
A plot of DFT-calculated transition-state energy-differences, Δ*E*(TS_H_-TS_C_), vs *F*_*Z*_, for the reaction of a model active species (Compound I) of Cytochrome P450, with propene, H_2_C=CHCH_3_. The respective oxidation products in the two EF-directions are shown beneath the plot. Reproduced from ref 29. Copyright 2004 American Chemical Society.

Thus, by using the TS-dipole moments for the two reactions (+15.09 D for *F*_Z_ > 0 and −9.87 D for *F*_Z_ < 0) and the respective OEEFs (*F*_Z_ = ±0.257 V/Å; i.e., ± 0.005 au), eq 1 predicts 18.6 kcal/mol as the stabilization of the TS for C=C activation (*F*_Z_ = −0.257 V/Å) vs 10.15 kcal/mol for the TS for C–H activation (*F*_Z_= +0.257 V/Å). Clearly, C=C epoxidation of propene is highly preferred under the OEEF.

## 5 Reactivity Patterns and Structural Effects of (O)EEFs

Since OEEFs lower the energy of charge-transfer (CT) states, application of OEEFs may also modify and control reaction mechanisms of nucleophile–electrophile pairs. A case in point is the oxidative addition of (PH_3_)_2_Pd to aryl halides and alkyl halides. Thus, we found that a moderate OEEF (0.15 V/Å) transforms the oxidative-addition reaction to a nucleophilic-displacement reaction.^48^ This was found for the reaction of (PH_3_)_2_Pd: with CH_3_Cl, in which the OEEF stabilizes the charge-transfer state [(CH_3_)_2_Pd^•+^CH_3_Cl^•–^] that correlates to the S_N_2 product [(CH_3_)_3_Pd^+^ + Cl:^–^].^48,49^

Having exemplified the impact of the main rules, let us describe additional examples from our work, some of which were experimentally tested. One concerns the enhancement of Diels–Alder reactivity,^1,4^ which was elegantly verified.^18,50^ Another study tested the impact of solvent screening, using a QM/MM/MD study of the Menshutkin reaction in three polar solvents.^3^ The latter study showed that **solvent-screening is imperfect**, and the OEEF in solution still confers significant barrier lowering. Another project (Scheme 1c) describes a design of a protein cage that possesses an OEF, and enhances the reactivity of molecules.^24^ Similarly, Arabi and Matta^51,52^ demonstrated the potency of OEFs to stimulate double-proton transfers between DNA bases. Finally, we describe the OEEF effect on the pyramidal inversion of ammonia, and proceed to experimentally tested destructions of Alzheimer’s peptide plaques by oscillating EEFs.^38,39^

Let us initially describe experimental studies, which tested the theoretical predictions of OEEF effects on the Diels–Alder reaction.^4^ We subsequently discuss the impacts of OEEF on the control of the *endo*/*exo* selectivity and enantioselectivity of this reaction. Then we display the recent achievement of enhanced reactivity of the Huisgen cycloaddition,^53^ in a multimolecular OEEF device. This will be followed by discussing other experimental studies of product-selectivity, in various reactions.^22,23^

### 5.1 The Diels–Alder Reaction

Figure 5 shows energy barriers and TS dipole moments for Diels–Alder reactions of cyclopentadiene and maleic anhydride under *F*_Z_ fields.^4^ The reaction axis (**RA**) Z is perpendicular to the two molecular planes, and the rate enhancement occurs along this single direction, which coincides with the direction of charge transfer. The common pattern of barrier lowering and barrier raising along the Z direction reappears; *F*_Z_ < 0 lowers the barriers, whereas *F*_Z_ > 0 raises the barriers. Furthermore, in the *F*_Z_ < 0 region the TS dipole moments increase by ∼4 D due to charge-transfer from cyclopentadiene to maleic anhydride. By contrast, *F*_Z_ > 0 raises the barriers and reduces the amount of charge transfer and hence also the TS dipole moment, which diminishes (e.g., 0.01 D for the TS of the *exo* isomer). The barriers behave as predicted by eq 1, in which barrier lowering is gauged by the increasing TS dipole, and vice versa for barrier raising.

**Figure 5:**
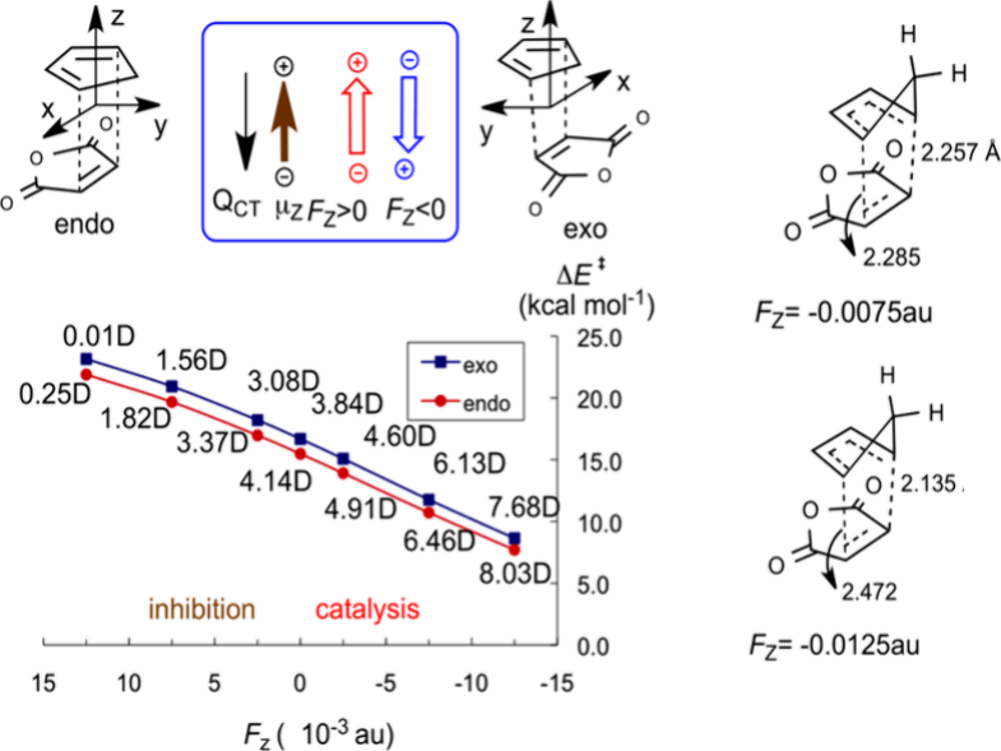
A computational study of the Diels–Alder reaction of cyclobutadiene and maleic anhydride in the *exo* and *endo* configurations. The **RA** is the *Z*-axis. The left-hand plot shows the energy barriers as a function of *F*_Z_. The transition-state dipole moments are marked on the lines (in debye (D) units). The structures on the right show two *endo* transition states under negative *F*_Z_ < 0 values. Reproduced from Figure 4 in ref 4 with permission. Copyright 2010 John Wiley and Sons.

As the structures on the right-side of Figure 5 reveal; increasing the negative *F*_Z_, makes the transition states significantly bond-nonsymmetric. When the reaction was computed in a solvent, **the highly distorted TSs collapsed to zwitterionic intermediates**,^4^ which relaxed to the cycloadducts. This prediction was verified experimentally, by Yang et al.^54^

Two more predictions on the same Diels–Alder reaction merit mention: thus, application of *F*_Y_ where Y is the axis between the planes of the reactants^4^ leads to *endo*/*exo* discrimination. In the *F*_Y_ < 0 direction, the field prefers *exo* over *endo*, and vice versa in the *F*_Y_ > 0 direction.^4^

Even more enticing, Figure 6 shows that applying *F*_X_ on the reaction of 1,1-disubstituted ethenes and cyclopentadiene yields *R or S enantioselectivity*, that depends on the *F*_X_ application. Thus, *F*_X_ > 0 prefers *R*, while *F*_X_ < 0 prefers *S*.^55,56^ As shown in Figure 6, the *R*/*S* discrimination in a given *F*_X_ depends on the dipole moment of the dienophiles. Thus, for 1,1-dicyanoethene, with the largest dipole moment in the series, Δ*E*(*R*/*S*) is 30 kcal/mol! Even for the nonpolar styrene, the discrimination exceeds 3 kcal/mol, hence ∼100% enantioselectivity. We emphasize that the high value of 30 kcal/mol for the computed Δ*E*(*R*/*S*) is the difference between **a destabilized TS and a stabilized one**. This difference simply depends on the product of the TS dipole moment and applied *F*_X_ (eq 1).

**Figure 6:**
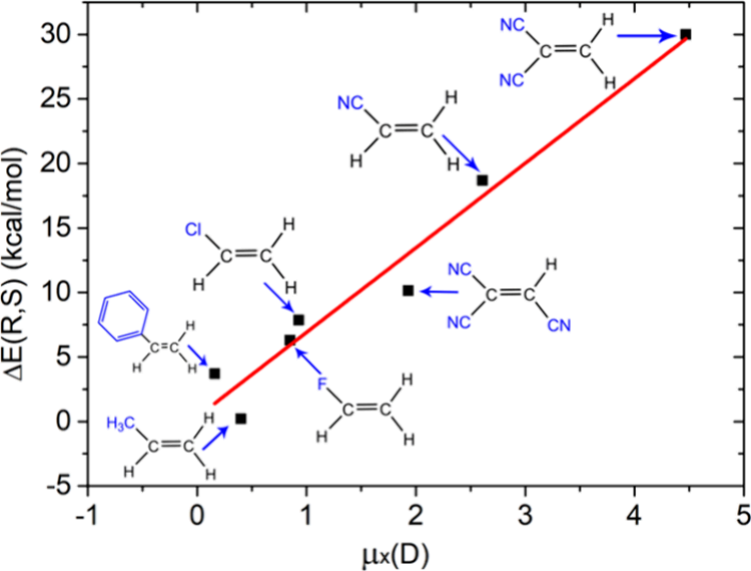
A study of computed chiral selectivity (ΔE­(*R*,*S*)) for various olefins with cyclopentadiene. Adapted from ref 55. Copyright 2018 American Chemical Society.

In conclusion, therefore, **changing the direction of the OEEF from Z to Y and X is predicted to control the rate and** ***endo/exo*** **selectivity, as well as** ***R*** **/** ***S*** **enantioselectivity**. Hence, when the reaction complex simultaneously samples EEFs along the X, Y, and Z components, this orientation **would enhance the rate and control the** ***endo/exo*** **selectivity as well as the** ***R*** **/** ***S*** **enantioselectivity of the reaction**. Although controlling this simultaneous-juxtaposition mode of the reactants is not straightforward, the ingenuity of experimental chemists may eventually overcome the difficulties of testing this idea.

### 5.2 How Does a Two-Directional EEF Affect Diels–Alder Reactions?

In **nonpolar** Diels–Alder reactions of diene-dienophile pairs, e.g., butadiene and ethylene, the electronic reorganization is cyclic. Thus, one anticipates a two-directional **RA**.^56^ Hence, **when the OEEF eventually reverses the relative donor–acceptor capabilities of butadiene-ethylene**, the two directions of the OEEF will lower the energy barrier for the reaction.^56^ While this is exciting, in polar cycloadditions like in Figure 5, the catalytic and inhibitory parts already span sizable barrier ranges along the **RA**. Hence, in reactions of good donor–acceptor pairs, the curving of the inhibitory portion to become rate-enhancing would require high *F*_Z_ > 0 values (>0.8 V/Å, Figure 5). On the other hand, for nonpolar pairs, OEF is likely to bend the inhibitory portion, in Figure 5, to a rate-enhancing one.^57^

## 6 What Is the Impact of Solvent Screening on OEEF?

When and how do EEFs lead to catalysis in the presence of solvents? This issue was addressed by using a combination of molecular dynamics (MD) simulations and quantum-mechanical/molecular-mechanical (QM/MM) calculations with OEEF. To assess the impact of solvent screening on OEEF effects, we selected the Menshutkin reaction of pyridine with CH_3_I, in three solvents of increasing polarity (chloroform, acetone, and acetonitrile).^3^ Figure 7 shows the energy profile in the presence of acetonitrile, without OEEF.^3^ The profile involves five species, and the rate-controlling barrier height is 18.9 kcal/mol.

**Figure 7:**
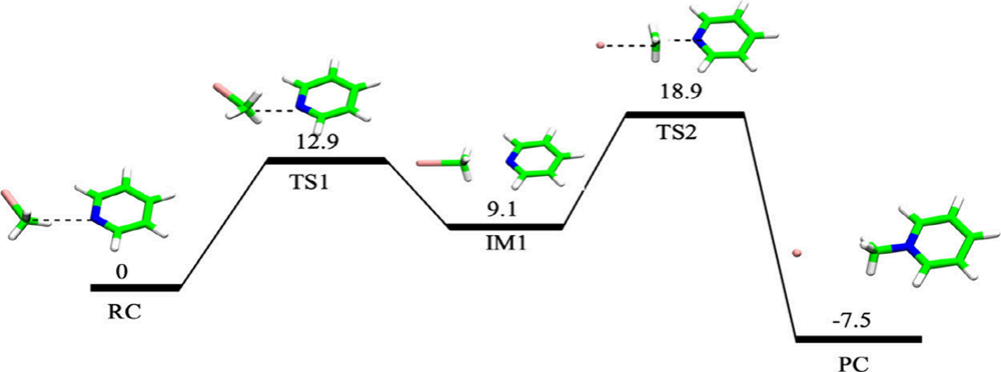
Energy profile of the Menshutkin reaction of pyridine and CH_3_I in acetonitrile (CH_3_CN). Adapted from Figure 2 in ref 3. Copyright 2020 American Chemical Society.

However, once an OEEF (0.5 V/Å) was applied, the remaining species were **RC** (**IM1**), **TS2**, and **PC**. Figure 8 shows the relative energies of **RC** and **TS′** in acetonitrile compared with the barrier (18.9 kcal/mol) in acetonitrile solvent without OEEF, i.e. **TS**_**no EEF**_ (i.e., **TS2** in Figure 7). Figure 8 shows that the OEEF lowers the barrier by 10.6 kcal/mol, **even in the presence of the “solvent screening”**. Using the other two polar solvents (acetone and chloroform) led to a barrier lowering of 12–12.6 kcal/mol.

**Figure 8:**
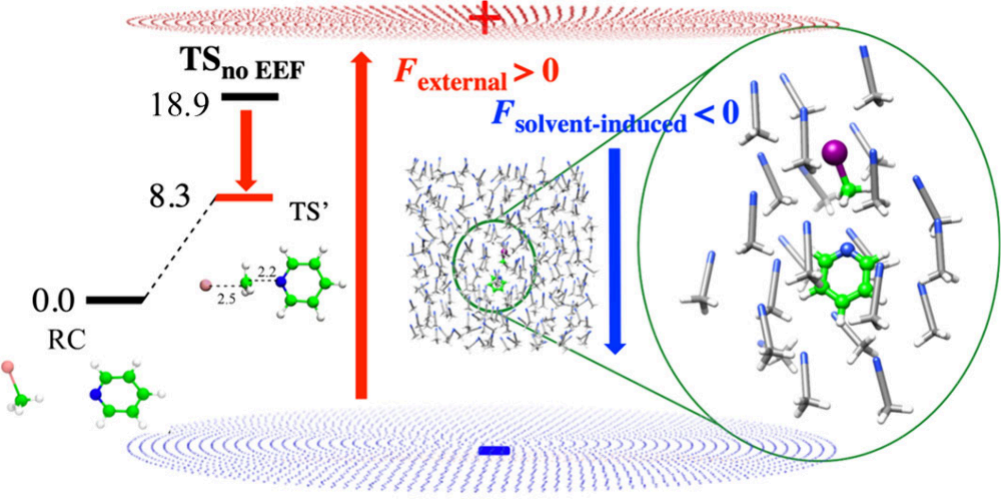
Menshutkin reaction in acetonitrile under OEEF= 0.5 V/Å (created by plates of point charges). The barrier on the left-hand side is 8.3 kcal/mol compared to the high barrier in the absence of OEEF (18.9 kcal/mol). The solvent screening of the molecular complex is shown on the right-hand side spheroid. The electric-field vector due to the screening solvent (*F*_solvent‑induced_) is drawn in blue alongside the applied OEEF (*F*_external_). Adapted from Figure 2 in ref 3. Copyright 2020 American Chemical Society.

Thus, the solvent molecules oppositely orient their dipoles vis-à-vis the external OEEF (*F*_external_), and this orientation screens the external field. Nevertheless, the calculations show that once *F*_external_ reaches 0.2 V/Å, **saturation sets in**, and no further solvent molecules get oriented by *F*_external_. This saturation occurs **since the unidirectional dipoles of the solvent molecules are mutually repulsive**. Therefore, fuller screening by more and more solvent molecules is prevented by the solvent­(molecule)–solvent­(molecule) repulsions. The solvent screening effect reaches saturation quickly, and the net *F*_external_ lowers the barriers of the reactions by 10.6–12.6 kcal/mol (in acetonitrile, acetone, and chloroform).^3^ As such, “solvent screening” of the OEEF is a misleading qualification. **The screening is incomplete, and rate enhancement by the OEEF remains highly significant in solutions!**

## 7 Structural Transformations Elicited by EEF/OEEF

(O)­EEFs can change the structures of molecules.^17^ One example is the *cis–trans*/*trans–cis* isomerization of azobenzene derivatives.^25^ Thus, using pressure,^58^ or scanning tunneling microscopy (STM) on a charged Au­(1,1,1) surface,^25^ led to reversible *trans–cis* isomerization of azobenzene derivatives. Our DFT calculations^17^ show that in the field-free situation, the *trans–cis* isomerization barrier is 39.2 kcal/mol. However, applying OEEF = 1.16 V/Å along the N=N bond lowers the barrier to 12.6 kcal/mol. This barrier accounts for the experimental observation of a switchable isomerization,^25^ which transpires by the OEEF effect.

Further along the structural line, we computed the effect of OEEF­(*F*_*Z*_) on ammonia. As shown in Figure 9, *F*_*Z*_ < 0 leads to a barrier-free inversion of the initial pyramidal structure (at *F*_*Z*_ = 0) into an inverted-structure having small LP–N–H angles (and an increased dipole moment).

**Figure 9:**
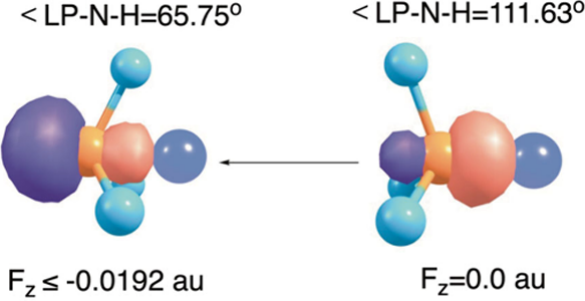
Applying on NH_3_ an *F*_*Z*_ < 0 along the lone-pair (LP) axis causes the ammonia to invert its structure, in a barrier-free fashion.

Another structural problem, which we computationally investigated, is the Cope rearrangement^59^ of semibullvalene. Using Cu^+^ as the source of the electrostatic field^60^ changes the energy profile^59^ from a single barrier to a stepwise reaction with two intermediate structures on both sides of the rearrangement barrier.

Finally, we investigated the destruction of a peptide plaque (10 peptides), which models plaques that develop during brain diseases.^38,39^ The zwitterionic peptides are drawn as arrows, where the heads are the negatively charged ends, while the other ends are the positively charged amino acids. These 10 peptides are arranged in Figure 10 as five head-to-tail (HT) dimers, which simulate the plaque. As such, the Os-EEF frequency plays a key role in decomposing these peptide dimers.^39^

**Figure 10:**
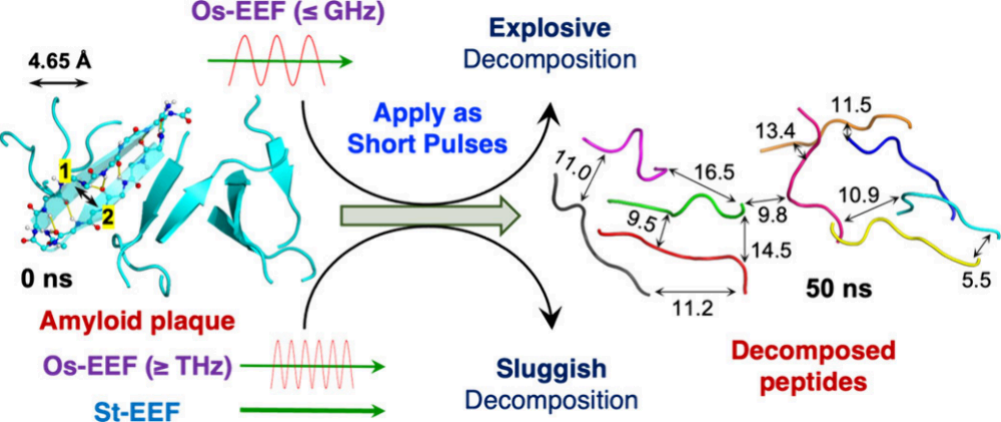
MD simulation results of applying an oscillating EEF (Os-EEF) and a static EEF (St-EEF) on the protein plaques. Adapted from ref 39. Copyright 2025 American Chemical Society.

Thus, using frequencies that are smaller or equal to 1 GHz, the plaque decomposes to individual peptides.^39^ In fact, **the plaque explodes** to individual peptides, wherein the average distances between peptides reach ca. 10–16 Å. The reason for this explosion is that in the GHz range or lower, one peptide in a dimer is stabilized by the Os-EEF, while its partner (having an opposite dipole) is destabilized.^38,39^ Hence the dimers in Figure 10 get instantly dispersed to long distances, like in an explosion.

At the bottom of Figure 10 are results for a St-EEF. Thus, when the HT dimers feel the St-EEF, these dimers decompose and the individual peptides *rearrange in a parallel mode, wherein the ensemble develops a huge dipole moment* (1130 D),^39^ which is stabilized by the St-EEF (eq 1). Consequently, the decomposition to individual peptides takes a long time, and the separated peptides exhibit short distances, ∼5 Å, followed by a fast reconstruction of the plaque. Under the Os-EEFs having frequencies ≥THz, the peptides are too heavy to follow the huge oscillation frequency of the field, and hence, the peptides behave as though being under the St-EEF, thus transforming to a parallel pairs ensemble with a large dipole moment. Subsequently the ensemble falls apart into individual peptides separated at ∼5 Å, leading to plaque reformation.^38,39^ Similar patterns of plaque disruptions by OEF have also been established experimentally.^61,62^

## 8 Experimental Reactivity Patterns

Other publications address issues, like spin-state selectivity in Cu^+^ catalyzed conversion of C + CH_4_ to ethene^60^ and in oxidations by nonheme oxo-iron complexes^63^ etc. Let us then discuss exciting experiments which demonstrated that the “EEF-reagent” produces versatile and panoramic chemistry that also differs from the corresponding thermal chemistry.

### 8.1 Experimental Testing of Predicted OEEF Effects on the Diels–Alder Reaction

The publication on OEEF effects on the Diels–Alder (DA) reaction was experimentally tested initially in 2016, using single diene and dienophile molecules, published in the now famous article in *Nature* ^50^ and later reviewed.^18^ As shown in Figure 11, the employed setup involved a gold tip holding the dienophile, whereas the diene was nestled on a gold electrode. In this manner, the electric field (voltage-source) **ran parallel to the RA**. Subsequently, the team increased the voltage difference (to Δ*V*_*b*_), and moved the gold tip toward the gold electrode. When the tip reached a distance of 1 nm, the DA adduct was formed and the observed tunneling current was increased. By disconnecting/reconnecting the gold tip from the product (see right-hand drawing), the team verified that the product was responsible for the observed current. As such, the study^18,50^ confirmed that the Diels–Alder product was formed because of the applied electric field.

**Figure 11:**
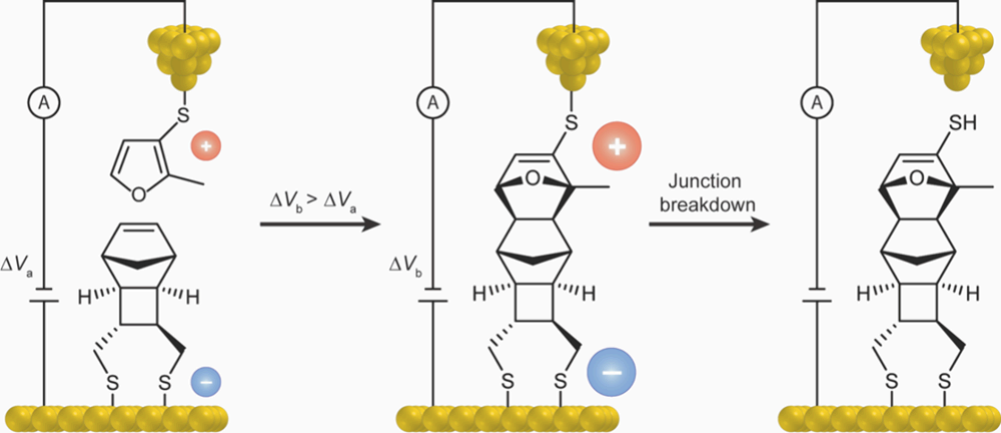
Experimental testing of the OEEF impact on a Diels–Alder reaction.^18,50^ The product (middle) is formed when the diene approaches the dienophile in the presence of a significant OEEF (Δ*V*_*b*_). The product formation completes the electric cycle and leads to a rise of the observed tunneling current. Disconnecting the product (on the right) stops the current. We are thankful to Nadim Darwish for the design of this figure.

Importantly, **in the absence of the OEEF**,^50^ the two molecules used in the experiment **do not produce much if any Diels–Alder adduct**. This makes a lucidly clear case: the OEEF is the root cause of the occurrence of this Diels–Alder reaction. **The product is an outcome of the OEEF application**! Other results by the same group on the influence of OEEF are summarized in a review.^18^ Similar OEEF impact was recently reported by Diez-Perez et al.^53^ for the Huisgen cycloaddition reaction in a multimolecular experiment.

Moreover, Yang et al.^54^ tested the OEEF effect on a Diels–Alder reaction and confirmed the impact of OEEF on the speed of the reaction, and **the formation of a zwitterionic intermediate at the highest used voltage**. In agreement with an early prediction by theory,^4^ Yang et al.^54^ verified the collapse of the zwitterions to the Diels–Alder *exo-* and *endo*-products.

### 8.2 Additional Experimental Verifications of the Impact of OEEF on Chemical Reactions

Kanan et al.^22^ produced an electric field, due to ion-separation in an electrochemical cell, and investigated the product selectivity for the reaction (see the middle of Figure 12). The positive electrode of the cell was polymer-coated, on top of which resides a rhodium catalyst. In this manner, there was no current in the cell, while an OEF existed due to the opposite-ion separation of the electrolyte.

**Figure 12:**
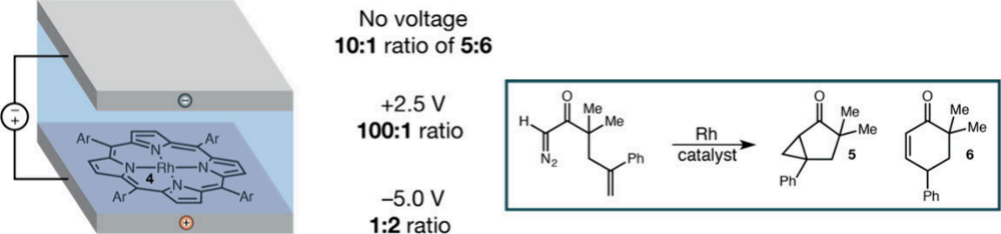
EEF-cell used in ref 22 is shown on the left-hand side. The lowest electrode of the cell is polymer coated, and as such, there is no current in the cell, only an EEF due to the separation of the ions of an electrolyte. The Rh catalyst rests on the coated electrode. Adapted from ref 22. Copyright 2013 American Chemical Society.

Thus, Figure 12 shows the carbenoid reaction that gives rise to products **5** and **6**. The product selectivity (**5:6**) at various voltages (OEFs) is shown in the middle. It is seen that **in the absence of OEF**, the Rh catalyst gives rise to **5**:**6** = 10:1. However, **in the presence of the OEF** (+2.5 V), **5**:**6** increases to 100:1. Finally, when the OEF is reversed (−5.0 V), so does the selectivity, which becomes 1:2. Apparently, *the OEF is more selective and subtle than the chemical rhodium catalyst*.

Recently, Matile et al.^23a^ and Matile, Wirth, et al.^23b^ have designed a new setup for producing EEF and “copying” it onto the reaction cell.^23b^ This setup uses surface charging of highly polarizable multiwalled carbon nanotubes (MWCNTs), which generate EEFs having directions that are controlled by the surface-charging voltage, as shown in Figure 13. At the bottom of the figure, we show one of the reactions which was catalyzed using this setup.

**Figure 13:**
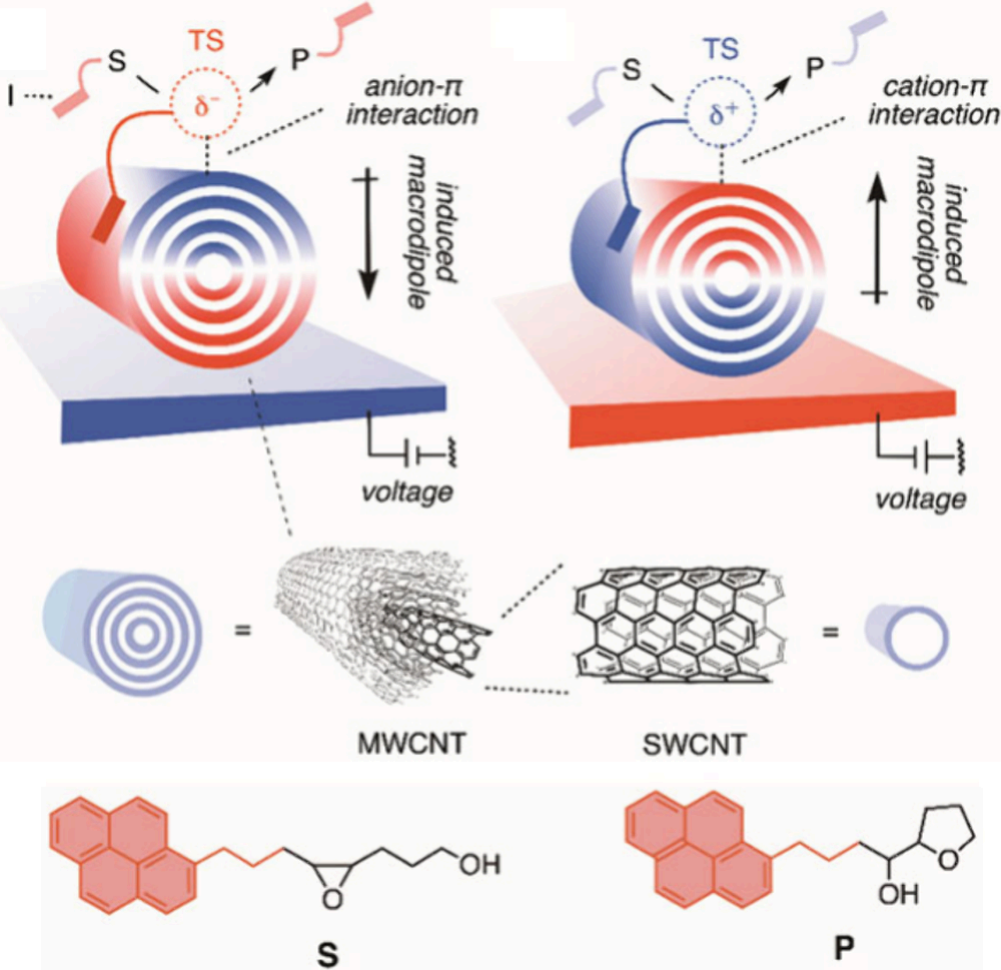
Devised OEF cell.^23b^ The device involves MWCNTs, which generate OEFs that are directed by the applied charging voltages. At the bottom of the figure is an example of a catalyzed nucleophilic attack which generates α-hydroxy-hydro-furan product (**S** → **P**). The brownish patches are pyrene rings, which serve as connectors that “copy” the EEF from the MWCNTs. Adopted from ref 23b. Available under CC-BY. Copyright Matile et al.

This method operates with a continuous-flow system, which, in principle, is quantitative. The yield determined by Wirth and Matile was 100 mM, which scratches the molar scale.

Recently, Matile et al.^64^ used the mono-terpene neryl chloride (**1** in Figure 14) in toluene solution. They observed the following cyclic product molecules, which depend on the direction of the OEF and on the direction and speed of the solvent flow. Thus, **in the absence of the OEF, the neryl chloride does not react.** When the OEF is applied, all the products are found to be cyclic; among them is the cyclohexenyl chloride (**2**).

**Figure 14:**
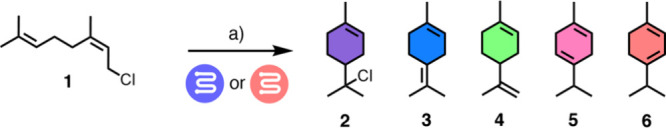
Products of neryl chloride (**1**) in toluene and an EEF. Underneath the arrows are directions of the solvent flow. Adapted from ref 64. Available under CC-BY. Copyright 2025 Matile et al.

The formation of **2** and other cyclized products underscores the presence of the OEF. Thus, the OEF causes **1** to lose a chloride ion, which is tossed away (this increases the dipole moment and is hence favored by the OEF) and leaves behind the initial neryl cation. The cation cyclizes and loses protons to form **3**–**6**. The return of the tossed-away chloride anion eventually forms chlorinated derivative **2**. In principle, the products may reach a molar scale using continuous flow setups.^23b,64^

In summary, OEFs lead to new products which are not obtained by standard thermal synthetic methods.^23b^ As such the EEF/thermal dichotomy is reminiscent of the photochemical/thermal dichotomy.^65^ To probe this dichotomy, one might consider studying sigmatropic shifts, forbidden cycloadditions, cyclization of dienes and trienes, etc. which possess specific stereochemical preferences.^65^ *Such studies, with OEEFs, may generate a fruitful methodology that leads to fast reactions, with products that may have unusual or desired stereochemical preferences.*

## 9 Concluding Remarks

This ACR article demonstrates that external electric fields, which can also be oriented (OEEFs), play roles as “reagents” that change structure and enhance reactivity. The reactivity and selectivity of these “(O)­EEF reagents” are higher than those obtained in thermal reactions, which are catalyzed, e.g., by transition metal catalysts.^22,23b^ Furthermore, because the electric field is a vector that has specific symmetry, these “reagents” are likely to generate new products and/or results that differ from the results of the thermal reaction. This is found indeed by experimentalists who are cited in this Account, e.g., by Kanan et al.^22^ and Matile et al.^23,64^

The methods are already approaching molar scalability,^23,53,64^ and their chemical importance will rise. The future of the OEF/OEEF “reagents” ultimately depends on the experimental community; already, it looks quite promising. **New methods for generating OEF sources** continue to appear, as in the recent invention of Zare et al.,^66^ who showed that tribo-charging by a rapid tape peeling generates a field of 1 V/Å, giving a new tool for EEF-driven chemistry. Thus, in the years to come, EEF/OEEF usage may impact chemical education and the art of making new molecules. While a computed reaction mechanism may not constitute a precise mechanism, still theory provides insights into experimental results and offers predictions, which can be further tested. The experiment–theory duo is a versatile combination!

## §

S.K.: Department of Chemical Sciences, Tezpur University, Napaam, Tezpur, Assam-784028, India
